# Supplementary material for: Self-Repairing Herpesvirus Saimiri Deletion Variants
Source: Viruses. 2022 Jul 13;14(7):1525. doi: 10.3390/v14071525 (PMC9320899; doi:10.3390/v14071525)
Supplement: Supplementary file 1 [file viruses-14-01525-s001.zip › viruses-1809795-supplementary.pdf]

Self-Repairing Herpesvirus Saimiri Deletion Variants

Ines Heyn, Linda Bremer, Philipp Zingler and Helmut Fickenscher *

S. Supplementary data

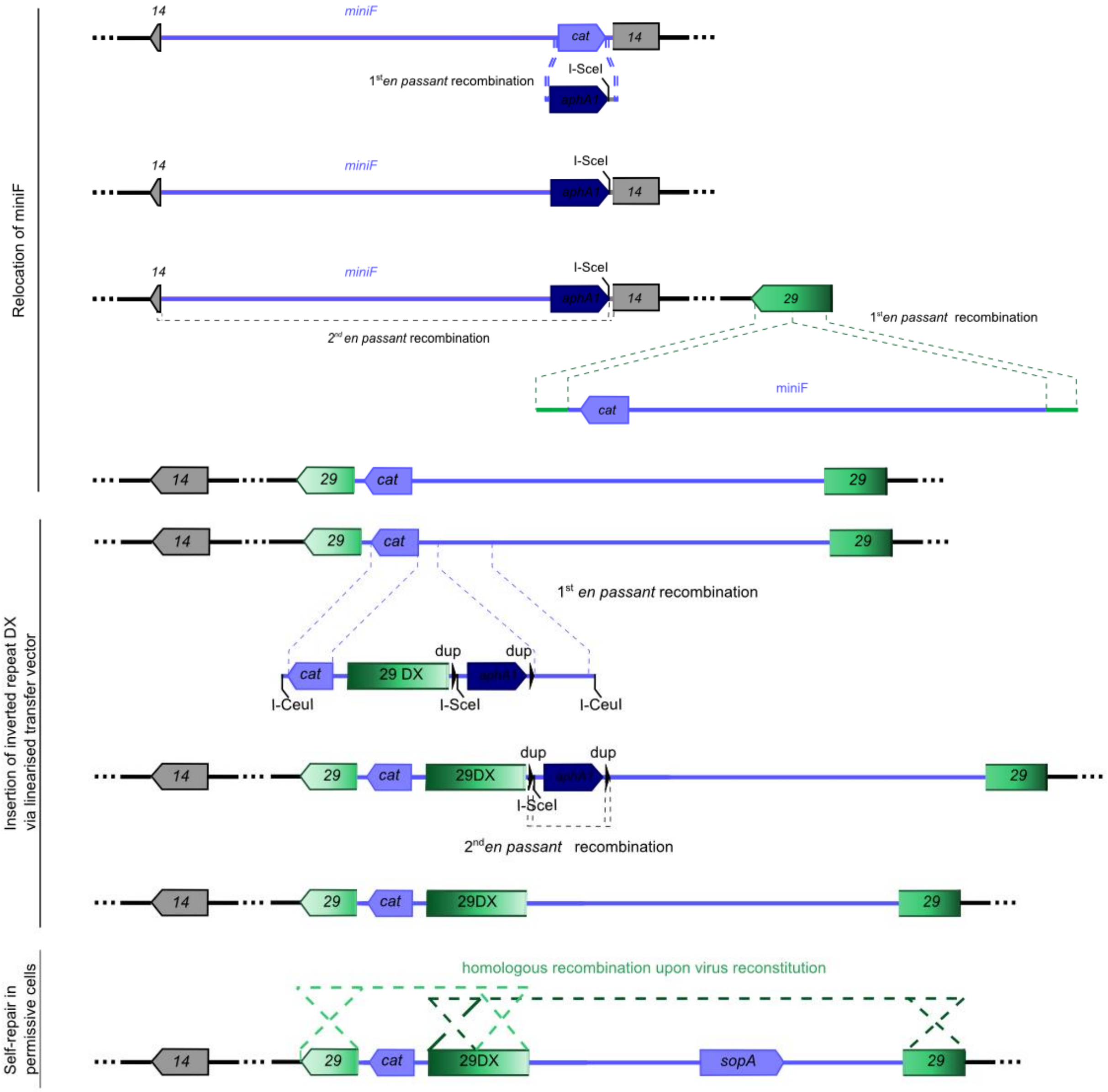

Figure S1. Scheme of complete procedure for generation of self-repairing HVS BAC variant WT29fDX. First, the chloramphenicol resistance gene *cat* is replaced via en passant mutagenesis with the kanamycin resistance gene *aphA1*. Second, simultaneously the miniF is inserted into *ORF29*, while the miniF is released via second en passant recombination from *ORF14*. *ORF14* is completely restored to function, while *ORF29* now is disrupted. After relocation, a previously generated transfer vector was linearized using I-CeuI restriction digest and used to insert inverted sequence duplications (DX) homologous to *ORF29*-segments up- and downstream of the miniF sequence (indicated by green color gradient). Upon reconstitution in permissive cells, homologous recombination can occur and the viral genome can self-repair *ORF29* and restore it to function.

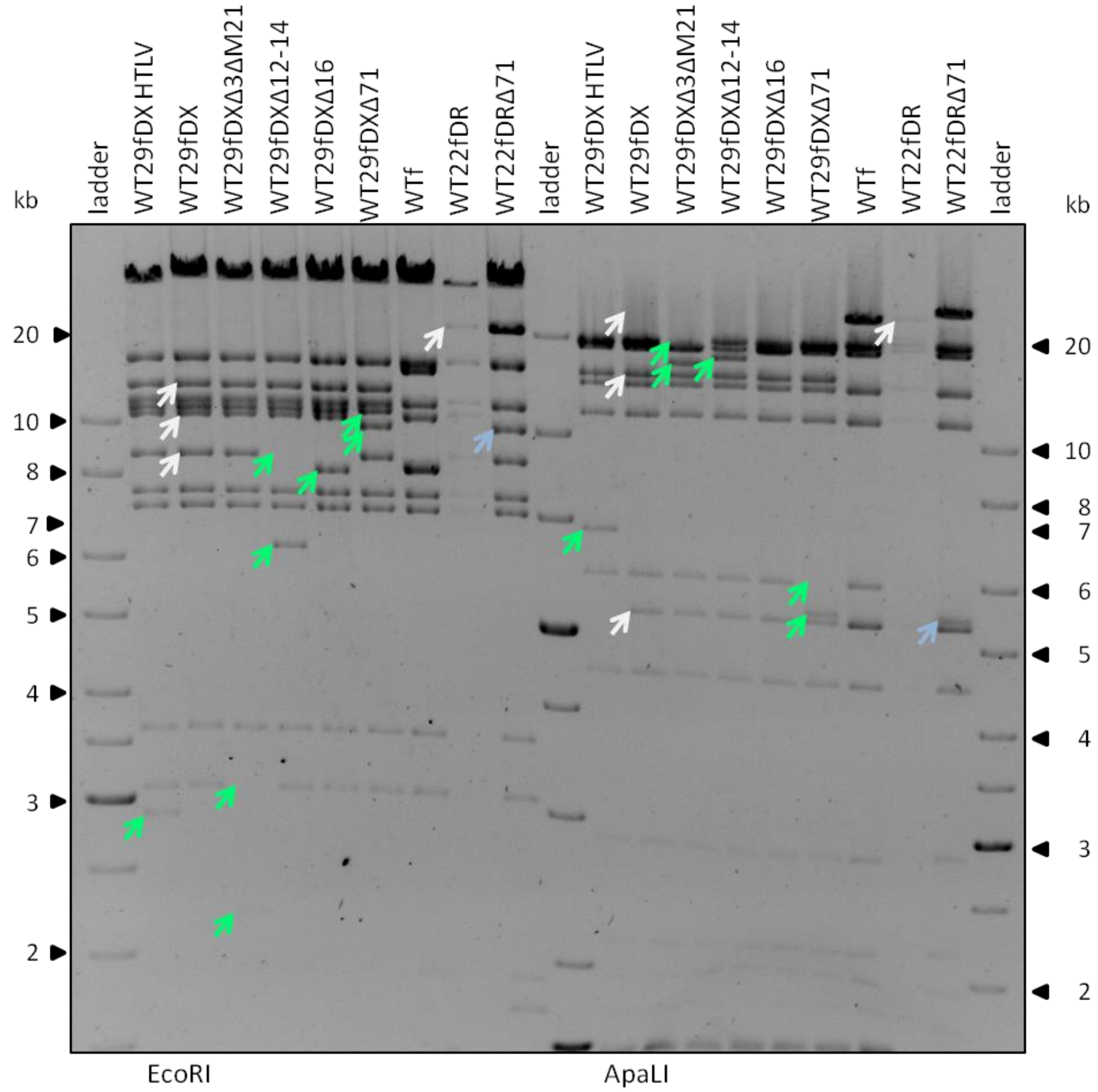

Figure S2. RFLP of indicated manipulated virus variants based on self-repairing BACs WT29fDX and WT22fDR, compared to the wildtype BAC WTf. Restriction digestion was performed using EcoRI and ApaLI, respectively, for 8 h at 37 °C. The gel electrophoresis was run at 50 V for 24 h on a 0.6 % agarose gel. White arrows indicate Band shifts from self-repairing variants compared to the WTf BAC band pattern, green arrows indicate changes of WT29fDX-based variants compared to the predecessor W29fDX, while light-blue arrows indicate band pattern changes of WT22fDRΔ71 compared to WT22fDR.

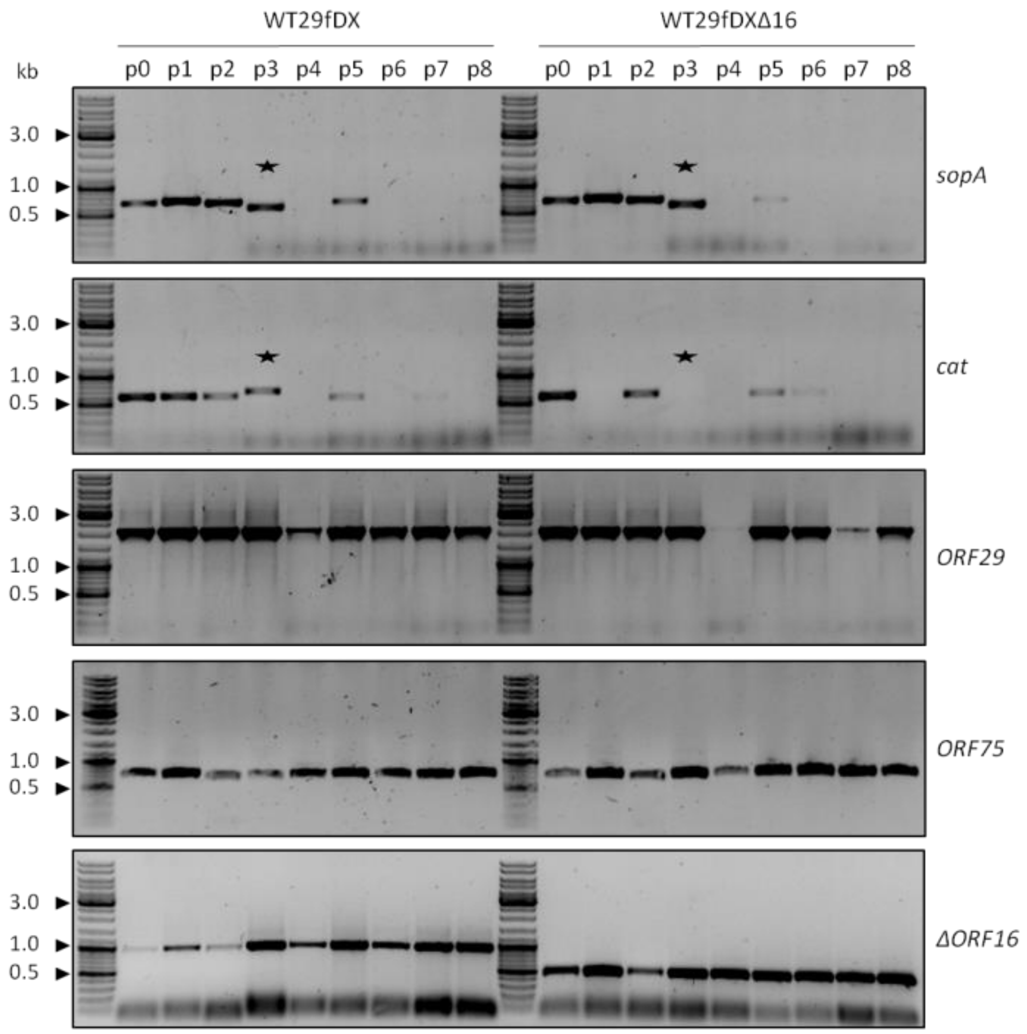

Figure S3. PCR-monitoring of the self-repair of WT29fDX and WT29fDXΔ16. Passaging differed from the other viruses by introducing a larger volume (3 ml) of infectious supernatant on permissive OMK cells and incubating it until complete lysis was reached. The star indicates a swap during application on the gel of passage 3 between *cat* and *sop* PCR samples for both viruses. The newer passaging method incubating a small volume of infectious supernatant for only 1 h on permissive cells resulted in a faster loss of the miniF sequence.

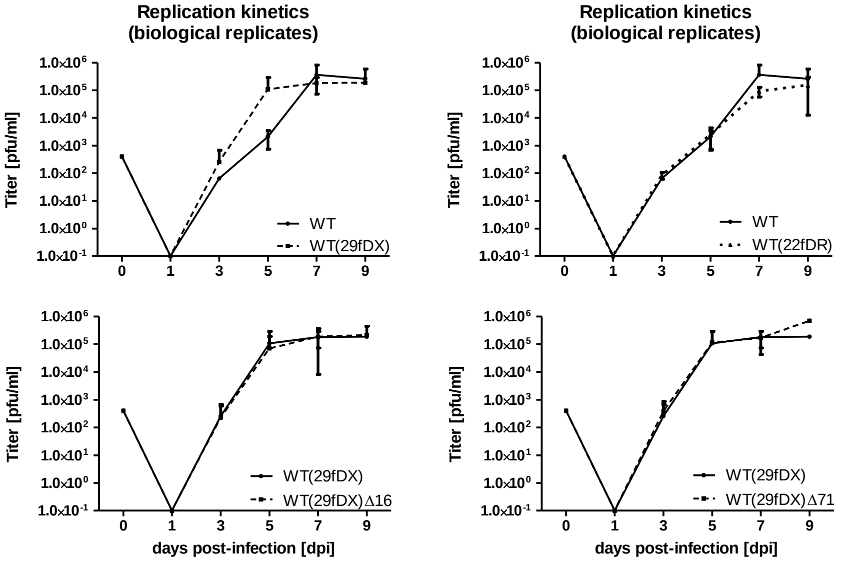

Figure S4. Exemplary normalized replication kinetics of generated virus variants. Each data point indicates three technical replicates of each three biological replicates. Error bars indicate standard deviation.

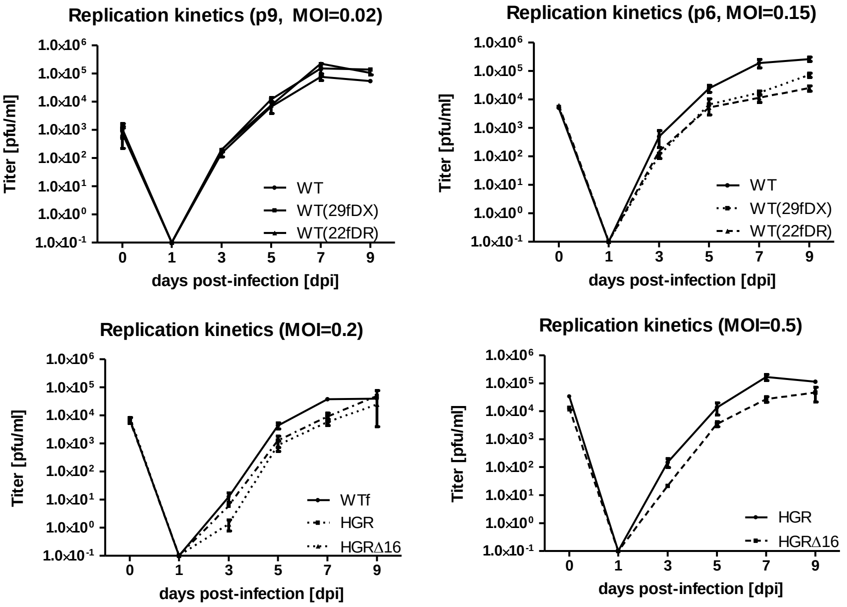

Figure S5. Examples for replication kinetics of different virus variants at different MOIs between 0.01 and 0.5. The higher the inocula, the shorter the infection period, since infection cannot develop successively but is complete in almost one round of release with no cells left to infect, resuling in lower endtiters. Inocula lower than 0.01 regularly result in high variations and, thus, higher standard deviations. The virus variants WT (wildtype), WTf (wildtype with miniF in ORF14), WT(29fDX) (self-repaired WT29fDX virus), WT(22fDR) (self-repaired WT22fDR virus), HGR (wildtype with additional eGFP under control of DOX-on system) and its corresponding ORF16 deletion variant.

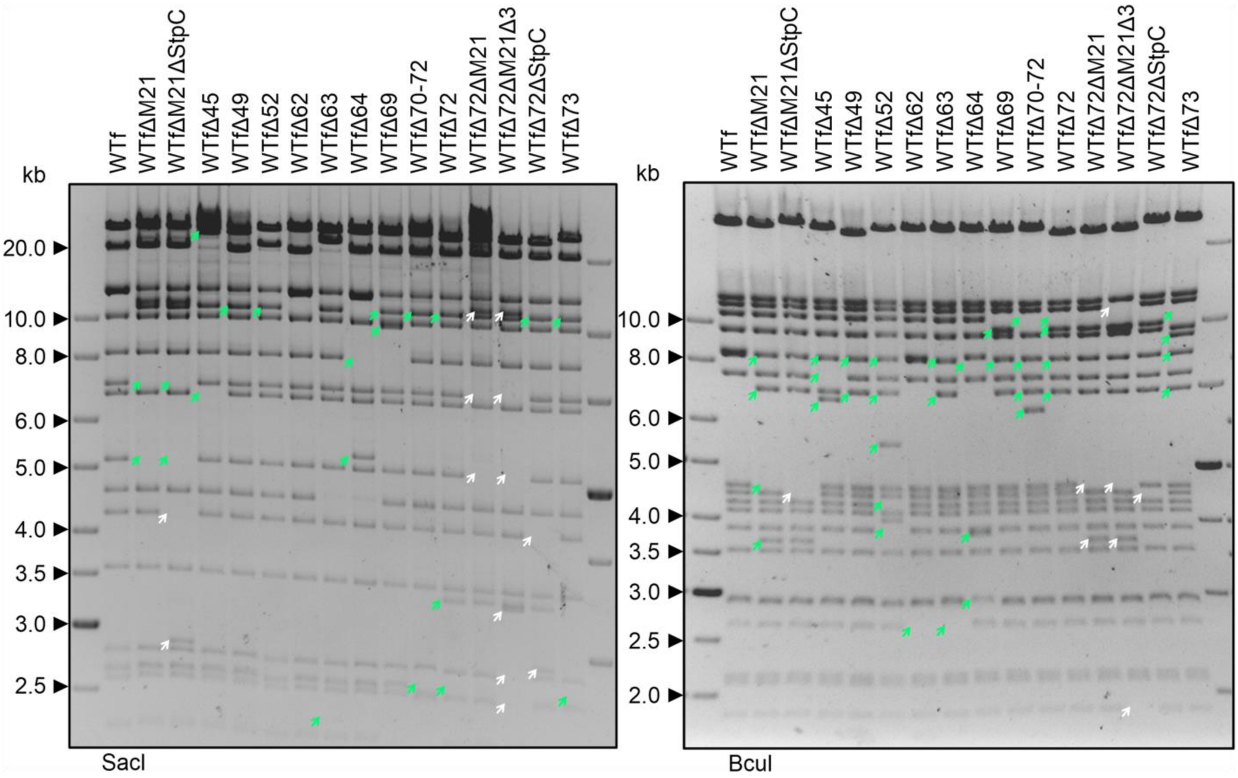

Figure S6. RFLP of indicated manipulated virus variants compared to the wildtype BAC WTf. Restriction digestion was performed using SacI and BcuI, respectively, for 8 h at 37 °C. The gelelectrophoresis was run at 50 V for 24 h on a 0.6 % agarose gel. Green arrows indicate the expected band-pattern changes of the deletion variant compared to the wildtype, white arrows indicate expected band shifts distinct from the predecessor deletion variant.

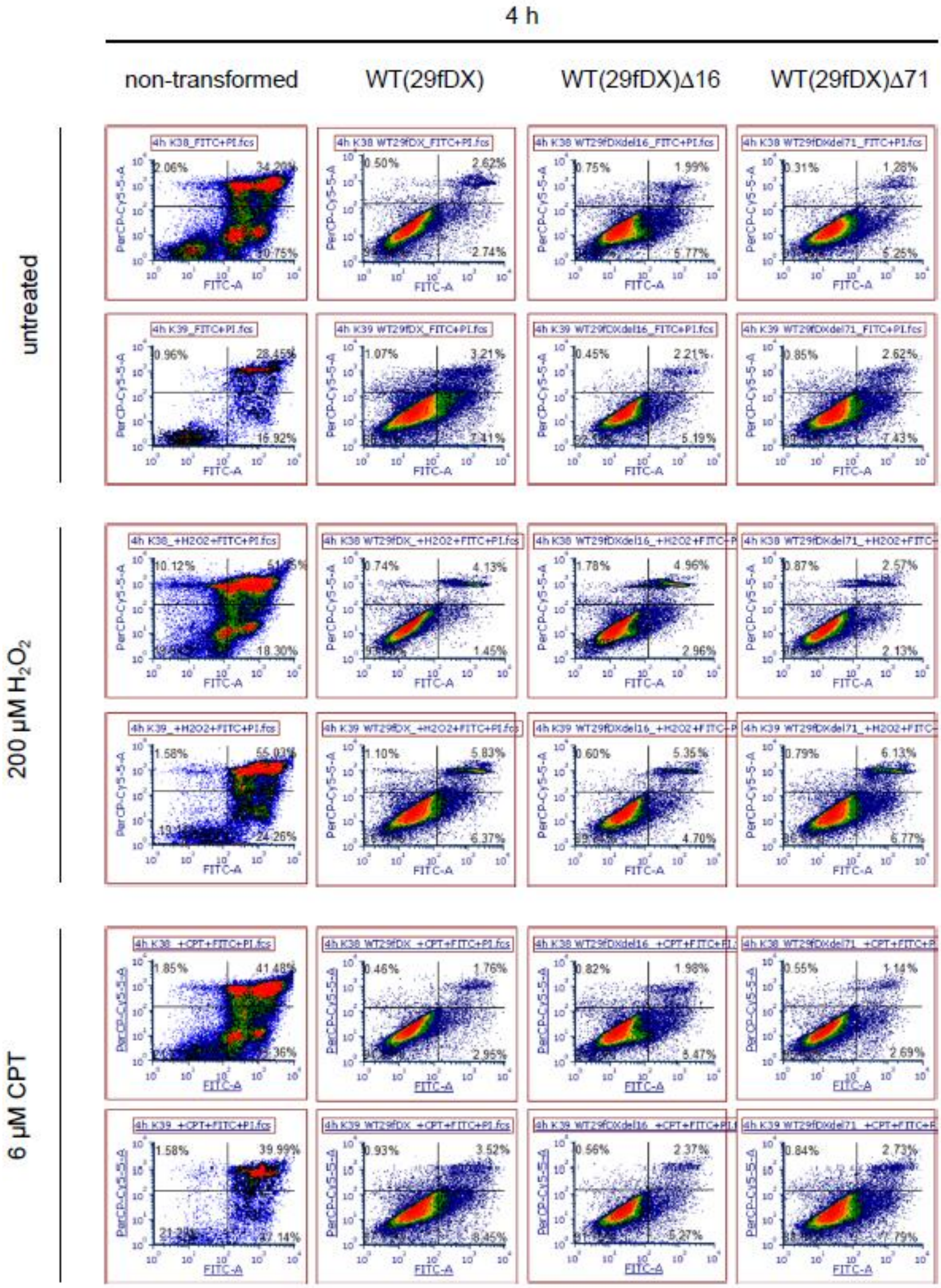
4 h
non-transformed
WT(29fDX)
WT(29fDX)Δ16
WT(29fDX)Δ71
untreated
200 μM H2O2
6 μM CPT
PerCP-Cy5-5-A
FITC-A

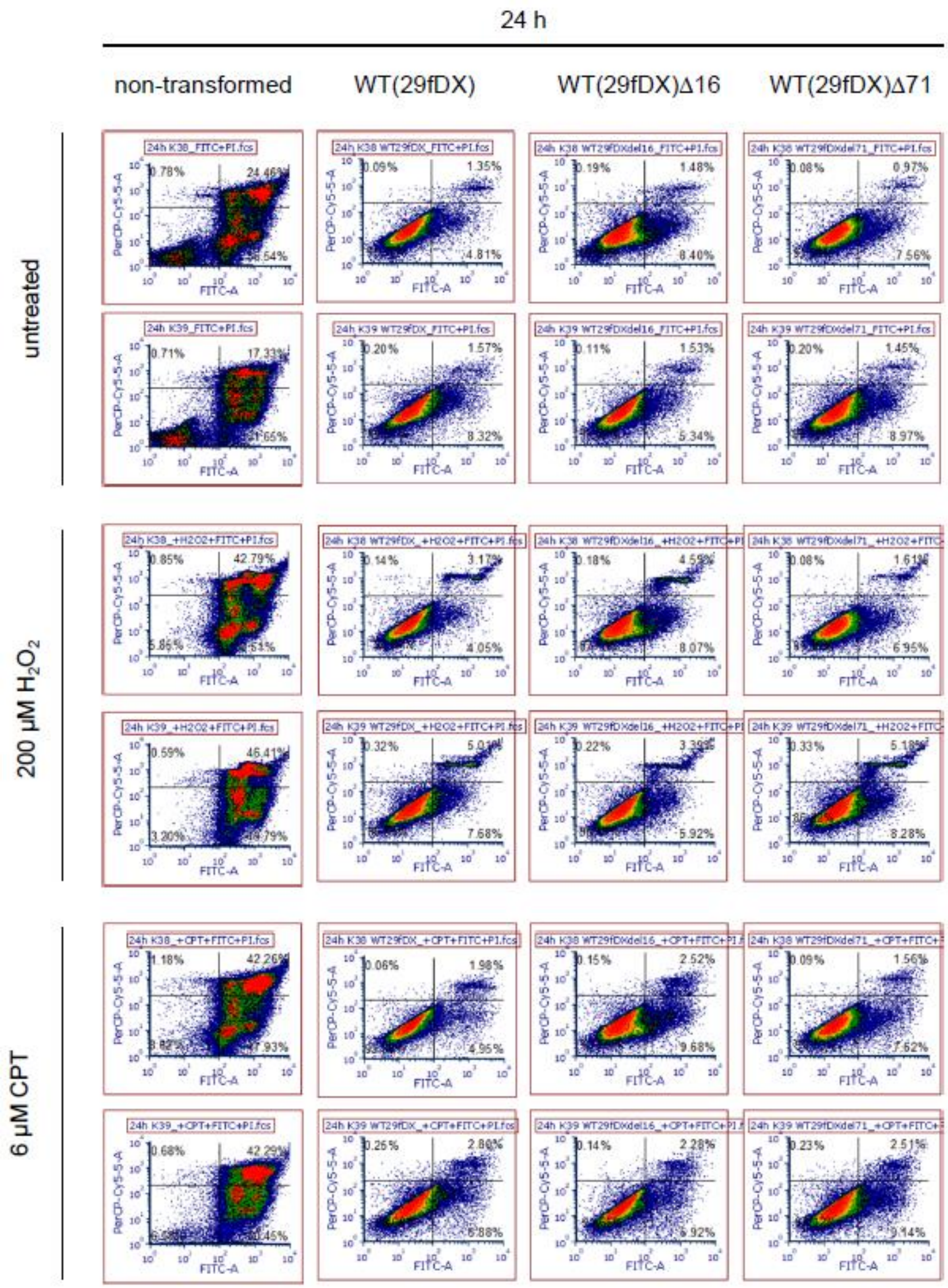

Figure S7. Apoptosis induction in 31-months-old HVS-transformed human T cells after 4 h and 24 h (continued). Density plots generated using FCS express 6. Hydrogen peroxide (H_2O_2) was applied to stimulate the intrinsic apoptosis pathway and camptothecin (CPT) to induce apoptosis via DNA damage. At 4 and 24 h after induction, the apoptosis rates were quantified using FITC-AnnexinV/ propidium iodide staining (Becton Dickinson).Viable, unstained cells presented in the lower left quadrant. Early apoptotic cells were marked by binding of FITC-Annexin to phospholipid phosphatidylserine and presented in the lower right quadrant. Propidium iodide (PI) positive cells marked dead cells and were located in the upper left quadrant. Late-apoptotic, double-positive cells were observed in the upper right quadrant. Image taken from [10].

Table S1. Average HVS-DNA copy numbers in 31-months-old, transformed human T cells of donor K39. DNA of 1 x 10^6 T cells was isolated and used to perform realtime PCR in relation to human GAPDH. Only technical replicates were possible due to low cell numbers.

**Cell line**	**Run 1**	**Run 2**	**Run 3**	***Mean***	**SD**
0618 K39 WT(29fDX)	247.38	59.90	85.17	***130.82***	*101.74*
0618 K39 WT(29fDX)Δ16	90.02	92.85	104.45	***95.78***	*7.65*
0618 K39 WT(29fDX)Δ71	99.97	82.81	98.56	***93.78***	*9.52*
